# Strychnia and Whiskey Hypodermically in Chloroform-Poisoning

**Published:** 1881-06

**Authors:** 


### STRYCHNIA AND WHISKEY HYPODERMICALLY IN CHLOROFORM-POISONING.

Dr. W. P. Nicolson, of Atlanta, Ga., reports in the *Southern Medical Record*, November, 1880, the case of a woman who took at once one ounce of chloroform. When seen, some hours afterwards by Dr. Nicolson, there was profound narcosis, with stertorous breathing; the cheeks and face were pallid and cold; there was entire loss of sensibility of the conjunctiva—the eyes remaining opened or shut according as they were left. After caffeine hypodermically had been used without effect, she was given hypodermically carbonate of ammonia, grs. ij, in whiskey ʒj, and strychnia sulphate gr. $\frac{1}{48}$. In about ten or fifteen minutes she moved her head and coughed, and expelled a quantity of mucus from her mouth, which had threatened to suffocate her. In about half an hour, half of the above dose—of whiskey and ammonia and the same amount of strychnia—were given; after which she rested quietly until morning, only occasionally being interrupted by vomiting. Her recovery was continuous. The points of interest were, the quantity of chloroform taken, the length of time before narcotic symptoms appeared, and the prompt action of the ammonia, strychnia and whiskey.

